# The m^6^A “reader” YTHDF1 promotes osteogenesis of bone marrow mesenchymal stem cells through translational control of ZNF839

**DOI:** 10.1038/s41419-021-04312-4

**Published:** 2021-11-12

**Authors:** Tao Liu, Xinfeng Zheng, Chenglong Wang, Chuandong Wang, Shengdan Jiang, Bo Li, Pengbo Chen, Wenning Xu, Huoliang Zheng, Runze Yang, Xingxu Huang, Xiaoling Zhang, Leisheng Jiang

**Affiliations:** 1 Spine Center, Xinhua Hospital Affiliated to Shanghai Jiaotong University School of Medicine, Shanghai, 200092 China; 2 Department of Orthopedic Surgery, The Affiliated People’s Hospital of Jiangsu University, Zhenjiang, 212002 Jiangsu China; 3 Department of Orthopedic Surgery, Xinhua Hospital Affiliated to Shanghai Jiaotong University School of Medicine, Shanghai, 200092 China; 4 School of Life Science and Technology, Shanghai Tech University, Shanghai, 201210 China

**Keywords:** Stem-cell biotechnology, Stem-cell differentiation

## Abstract

*N*6-methyladenosine (m^6^A) is required for differentiation of human bone marrow mesenchymal stem cells (hBMSCs). However, its intrinsic mechanisms are largely unknown. To identify the possible role of m^6^A binding protein YTHDF1 in hBMSCs osteogenesis in vivo, we constructed Ythdf1 KO mice and showed that depletion of Ythdf1 would result in decreased bone mass in vivo. Both deletion of Ythdf1 in mouse BMSCs and shRNA-mediated knockdown of YTHDF1 in hBMSCs prevented osteogenic differentiation of cells in vitro. Using methylated RNA immunoprecipitation (Me-RIP) sequencing and RIP-sequencing, we found that ZNF839 (a zinc finger protein) served as a target of YTHDF1. We also verified its mouse homolog, Zfp839, was translationally regulated by Ythdf1 in an m^6^A-dependent manner. Zfp839 potentiated BMSC osteogenesis by interacting with and further enhancing the transcription activity of Runx2. These findings should improve our understanding of the mechanism of BMSC osteogenesis regulation and provide new ideas for the prevention and treatment of osteoporosis.

## Introduction

Bone size and shape are precisely modeled and remodeled throughout life to ensure the structure and integrity of the skeleton [1]. Osteogenesis of bone marrow mesenchymal stem cells (BMSCs) and its underlying mechanisms are essential issues of bone formation and remodeling. Defective differentiation and dysfunction of BMSCs contribute to age-related changes and diseases such as sarcopenia and osteoporosis [2]. BMSCs are also regarded as relevant targets for therapies aiming to enhance bone regeneration.

BMSCs display remarkable plasticity and can undergo differentiation into different lineages, including adipocyte and osteoblast lineages in response to lineage-specific inducers in vitro or the bone marrow niche in vivo [3]. The balance of BMSC differentiation between osteogenic and adipogenic cell lineages is regarded as an important prerequisite for bone mass maintenance, and key transcription factors such as Runx2, Osterix, and activating transcription factor 4(Atf4), play decisive roles in BMSC osteogenic differentiation [4, 5]. Recent studies have revealed that the osteogenic differentiation of BMSCs is dependent on a network of transcription factors [6], although how the related transcription factors work together in this network is still unclear. Moreover, how the transcription factors themselves are regulated in many layers of control during BMSC osteogenesis is also not fully understood.

Recently, the chemical modification of RNAs has emerged as a new layer of epigenetic regulation [7]. To date, more than 170 types of RNA modification in mRNA, tRNA, rRNA, small nuclear RNA (snRNA), small nucleolar RNA (snoRNA), and long noncoding RNA (lncRNA) have been identified [8, 9]. Among them, *N*6-methyladenosine (m^6^A) is the most prevalent messenger RNA (mRNA) modification in mammalian cells [10]. It is dynamically deposited, removed, and recognized by m^6^A methyltransferases, demethylases, and m^6^A-specific binding proteins [11]. Although m^6^A has been implicated in various biological processes, including BMSC differentiation, its exact role and mechanism during BMSC osteogenesis require further elucidation.

Here, we observed that YTHDF1, an m^6^A binding protein, is indispensable for BMSC osteogenic differentiation. The downstream target of YTHDF1, ZNF839 (a novel zinc finger protein), was post-transcriptionally upregulated by YTHDF1 in an m^6^A-dependent manner both in vitro and in vivo. We also discovered that ZNF839 served as a co-activator of Runx2 and potentiated BMSC osteogenesis via interacting with it. These findings should improve our understanding of the mechanisms involved in the regulation of BMSC osteogenesis and provide new ideas for the prevention and treatment of osteoporosis.

## Results

### YTHDF1 is related to osteoporosis and its expression increases in human BMSCs (hBMSCs) during osteogenic differentiation

**Fig. 1:**
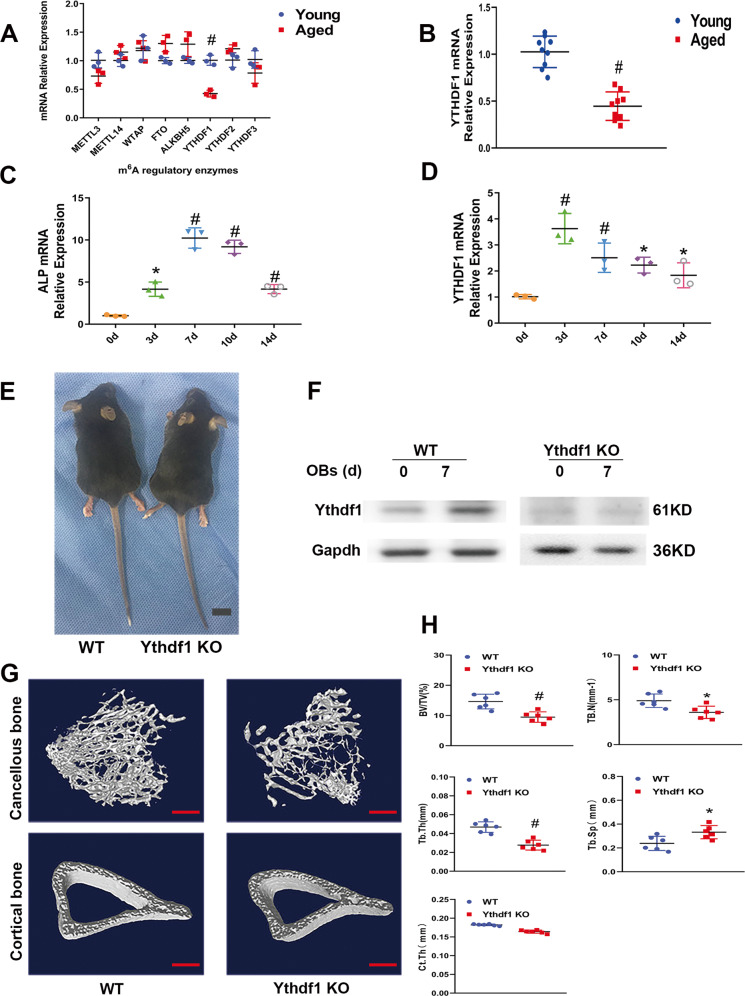
YTHDF1 is related to osteoporosis, and its expression increases in hBMSCs under osteogenic differentiation. **A** RT-PCR analysis of the changes in m^6^A regulatory enzyme levels in hBMSCs from young healthy male individuals and aged osteoporotic male patients. All experiments were independently performed in triplicate. (Paired *t* test, *n* = 3, ^#^*p* < 0.01). **B** YTHDF1 mRNA expression was detected in hBMSCs from aged osteoporotic patients and hBMSCs from young healthy male individuals. All experiments were independently performed in triplicate. (Paired *t* test, *n* = 9, ^*#*^*P* < 0.01). Horizontal lines = mean expression level. **(C**, **D)** RT-PCR analysis of the mRNA expressions of ALP and YTHDF1. hBMSCs were cultured in an osteogenic medium, and total RNA was collected at 0, 3, 7, 10, and 14 days; GAPDH was used as the internal control. All experiments were independently performed in triplicate. (Paired *t* test, *n* = 3, *P < 0.05, ^*#*^*P* < 0.01 versus day 0). **E** Gross images of 12-week-old Ythdf1 KO mice and their WT littermates. Images are representative of 6 mice per genotype; scale bar = 5 mm. **F** Ythdf1 deletion was confirmed with Western blot in BMSCs derived from KO mice, and the Ythdf1 protein expression was observed in WT BMSCs after 7 days of osteogenesis (OB: osteoblastic induction). All experiments were independently performed in triplicate. (*n* = 3 per group). **G**, **H** µCT analysis of the proximal tibia from 12-week-old male WT and Ythdf1 KO mice; scale bar = 500 µm. BV/TV: bone volume per tissue volume, TB.N: trabecular number, TB.Th: trabecular thickness; Tb.Sp: trabecular separation; Ct.Th: cortical bone thickness. All experiments were independently performed in triplicate. The results are presented as the mean ± S.D. (*n* = 6 per group, significant difference by Student *t* test; *P < 0.05, ^*#*^*P* < 0.01).

To understand the possible role of m^6^A in hBMSC osteogenesis as well as in osteoporosis, we detected the mRNA level of m^6^A regulatory enzymes in hBMSCs obtained from three young healthy male individuals (age range: 24–29 years old, average: 26.7 years) and three aged osteoporotic male patients (age range: 71–77 years old, average: 74.8 years) using real-time polymerase chain reaction (RT-PCR). Compared with other m^6^A regulatory enzymes, the level of YTHDF1 mRNA decreased dramatically with age (Fig. 1A). We then further expanded the sample number to 9 cases (age range: 20–30 years old, average: 26.4 years) VS (age range: 70–80 years old, average: 75.2 years) in each group and confirmed the significant difference in the level of YTHDF1 mRNA between the two groups (Fig. 1B). These results suggest that YTHDF1 may be involved in the pathogenesis of osteoporosis.

We then determined the mRNA expression profiles of YTHDF1 in hBMSCs cultured in an osteogenic medium. During osteogenic differentiation, YTHDF1 mRNA expression showed a similar but earlier distribution of alkaline phosphatase (ALP). The level of YTHDF1 mRNA increased and peaked on day 3 and then slightly decreased until day 14. Meanwhile, the cells exhibited elevated ALP expression with a peak that lagged slightly behind that of YTHDF1 on day 7. Then, the level of ALP mRNA gradually decreased on day 10 and further slightly decreased on day 14 (Fig. 1C, D). These data demonstrate that the YTHDF1 gene is expressed during hBMSC osteogenic differentiation in a time-dependent manner.

We further observed the potential role of Ythdf1 in BMSCs lineage allocation and bone metabolism in Ythdf1 Knockout (KO) mice. Ythdf1 KO mice were viable and were of similar size compared with their control littermates at 12 weeks of age (Fig. 1E). Western blot analysis confirmed the successful deletion of Ythdf1 in Ythdf1 KO mouse-derived BMSCs and the increased expression of Ythdf1 protein during osteogenesis in wild-type (WT) mouse-derived BMSCs (Fig. 1F). The micro-computed tomography (µCT) analysis of the trabecular bone from proximal tibia metaphysis revealed that Ythdf1 KO mice had lower overall bone mineral density (BMD) than WT mice (Fig. 1G), as evidenced further by diminished trabecular bone volume over total volume (BV/TV), trabecular number (Tb.N) and trabecular thickness (Tb.Th), whereas trabecular separation (Tb.Sp) was higher in Ythdf1 KO mice (Fig. 1H). In addition to the trabecular phenotype, Ythdf1 KO mice exhibited a mildly decreased thickness of cortical bone compared with WT mice, as evidenced by cortical bone thickness (Ct.Th) showed in Fig. 1H.

### YTHDF1 is responsible for the osteogenic potential of hBMSCs

**Fig. 2:**
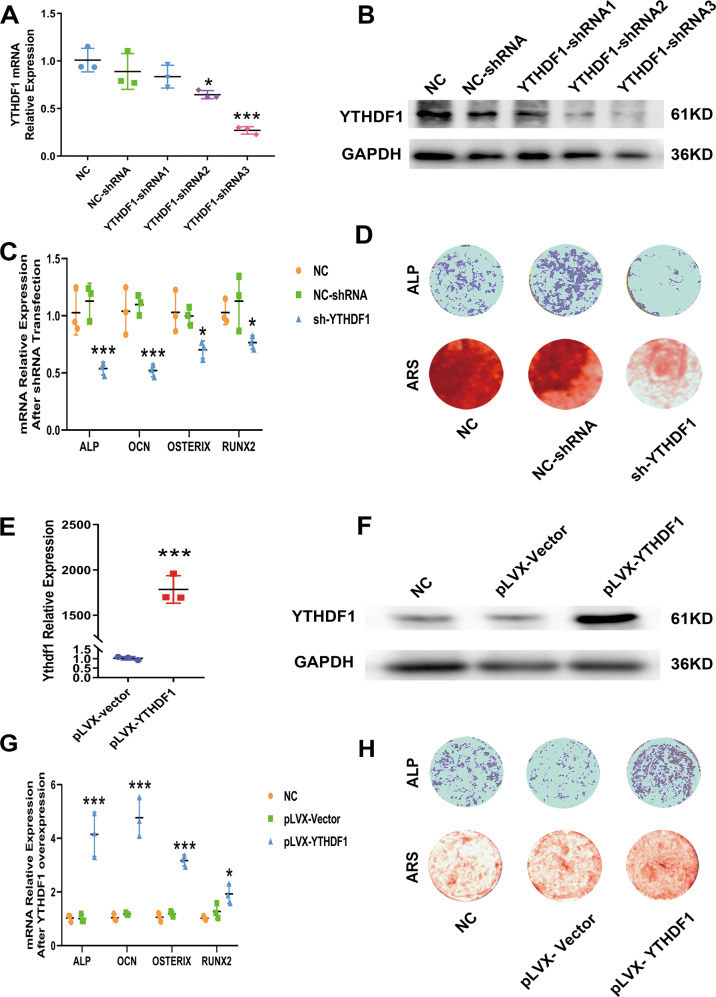
Effect of loss-and gain-of-YTHDF1 function on hBMSCs osteogenesis. **A**, **B** YTHDF1 mRNA and protein expression levels were detected 72 h after transfection of shRNAs in hBMSCs. All experiments were independently performed in triplicate (Paired *t* test, *n* = 3, *P < 0.05; ***P < 0.01 versus NC or NC-shRNA transfected samples). **C** mRNA expression levels of ALP, OCN, OSTERIX, and RUNX2 were all detected using RT-PCR at 72 h after osteogenic induction. All experiments were independently performed in triplicate (paired *t* test, *n* = 3, *P < 0.05, ***P < 0.01 versus NC or NC-shRNA transfected samples). **D** ALP and Alizarin Red staining were performed to detect osteogenesis of hBMSCs with shRNA3 after transfection for 7 or 14 days. **E**, **F** YTHDF1 mRNA expression and protein levels detected at 72 h after transfection of pLVX-YTHDF1. All experiments were independently performed in triplicate (Paired *t* test, *n* = 3, ***P < 0.01 compared with the pLVX-Vector group). **G** mRNA levels of ALP, OCN, OSTERIX, and RUNX2 were detected 72 h after osteogenic induction. All experiments were independently performed in triplicate (Paired *t* test, *n* = 3, *P < 0.05, ***P < 0.01 versus NC or pLVX-Vector transfected samples). **H** ALP and Alizarin Red staining after transfection with pLVX-YTHDF1 when cultured in osteogenic induction medium.

To determine the role of YTHDF1 in hBMSC osteogenic differentiation, we conducted loss-/gain-of-function experiments in hBMSCs. YTHDF1 expression was suppressed using shRNAs. The knockdown efficiency of three shRNAs was evaluated, and the YTHDF1-shRNA3 with the best efficiency was used in the following experiments (Fig. 2A, B). YTHDF1 knockdown led to a limited decrease in the mRNA expression of RUNX2, OSTERIX, ALP, and osteocalcin (OCN) in hBMSCs 72 h after osteogenic induction (Fig. 2C). Weakened intensities of ALP and Alizarin Red staining (ARS) were also shown in YTHDF1 knockdown hBMSCs compared with the control group (Fig. 2D). YTHDF1 overexpression was achieved by transfection of pLVX-YTHDF1 into cultured hBMSCs, and elevated YTHDF1 mRNA and protein expression levels were verified by PCR and Western blot analysis 72 h after transfection (Fig. 2E, F). At 24 h after pLVX-YTHDF1 transfection, hBMSCs were osteogenically induced, and cells transfected with pLVX-YTHDF1 showed enhanced intensities of ALP and ARS compared with the control group, with significantly higher mRNA levels of osteoblast-specific genes 72 h after osteogenic induction (Fig. 2G, H).

Elevated Ythdf1 mRNA and protein expression were also verified after pLVX-Ythdf1 transfection in mouse BMSCs (Fig. S1A, B). Consistent with the results of hBMSCs, the overexpression of Ythdf1 enhanced the osteogenic potential of mouse BMSCs, as indicated by ALP, ARS staining, and the expression levels of key osteoblast-specific gene mRNAs detected by PCR (Fig. S1C and D). However, BMSCs derived from Ythdf1 KO mice showed retarded osteogenesis (Fig. S1E, F). Both loss- and gain-of-function experiments in hBMSCs and mouse BMSCs confirmed that Ythdf1 is crucial to the osteogenesis of BMSCs.

### Me-RIP-Seq and RIP-Seq identified ZNF839 as a YTHDF1 downstream target

**Fig. 3:**
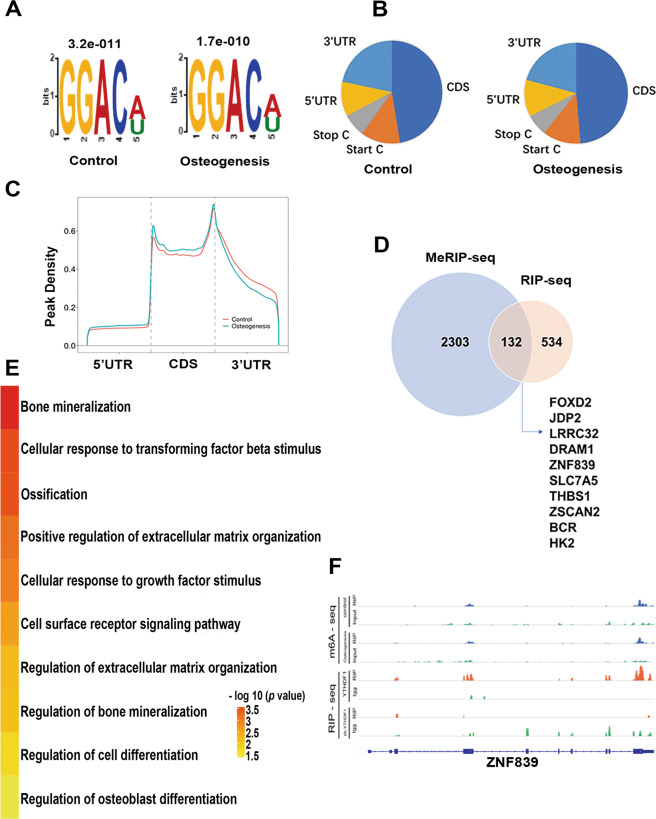
MeRIP-Seq and RIP-Seq identify ZNF839 as a target of YTHDF1. (**A**) Conserved motif identified with m^6^A-containing peaks, (**B**) Pie chart depicting the distribution with different gene contexts between YTHDF1 knockdown and control hBMSCs, and (**C**) Distribution of m^6^A-containing peaks across the length of mRNA. 5’-UTR, CDS, and 3’-UTR were each binned into 100 segments, and the percentage of m^6^A peaks that fall within each bin was determined. (**D**) Overlap of YTHDF1-binding peaks and m^6^A-containing peaks. (**E**) Representative GO terms of the biological process categories enriched in transcripts with YTHDF1, binding, and m^6^A peaks. GO analysis was performed using the DAVID bioinformatics database. (**F**) MeRIP-Seq demonstrating ZNF839 has a highly enriched and specific m^6^A peak near its translation stop codon.

hBMSCs that underwent 3 days of osteogenic induction and untreated control cells were subjected to Me-RIP sequencing (m^6^A-seq) assay to explore the profile of the m^6^A modification in untreated hBMSCs and the changes in this modification in hBMSCs during osteogenesis. More than 13,815 and 16657 m^6^A peaks were identified from the m^6^A-seq libraries generated from the osteogenic induction and control groups, corresponding to 8012 and 8729 transcripts, respectively. We also searched for consensus motifs and identified that the GGAC sequence, the most common m^6^A motif, was significantly enriched in the m^6^A peaks (*p* < 1.7e–10 for induced hBMSCs and *p* < 3.2e–11 for control cells) (Fig. 3A). To confirm the preferential localization of m^6^A in transcripts, we categorized the m^6^A peaks based on gene annotations and nonoverlapping segments as CDS (46.6%–50.7% of total peaks), Start C (12.4–10.9% of total peaks), Stop C (7.2–6.3% of total peaks), 5’-UTR (12.3–10.7% of total peaks), and 3’-UTR (21.6–21.4% of total peaks) (Fig. 3B). An analysis of the relative positions of m^6^A peaks in mRNA revealed that they were especially enriched in the vicinity of the stop codon in 3’-UTR region (Fig. 3C). Considering that dynamic m^6^A regulation plays a role during osteogenic differentiation, only m^6^A peaks in which the abundance changed upon osteogenesis were considered authentic m^6^A peaks. A total of 2303 genes with altered m^6^A peak numbers (*p* < 0.00001) were detected for further study.

Given that our m^6^A mapping method identified all m^6^A modified mRNAs but not those exclusively bound by YTHDF1, we sequenced the RNA obtained from the immuno-purified complex of YTHDF1 (RIP-Seq) to reveal YTHDF1-bound mRNAs to further identify its direct targets. With YTHDF1 knockdown cells used in this step, genes with a decreased binding affinity to YTHDF1 were the targets of interest. In total, 534 genes downregulated by sh-YTHDF1 in RIP-Seq were detected. To gain insights into the potential function of these genes bound to YTHDF1, we performed Gene Ontology (GO) enrichment analysis on these YTHDF1 target mRNAs with m^6^A peaks and observed that they are involved in various functions, including regulation of cell differentiation, regulation of osteoblast differentiation, and regulation of bone mineralization (Fig. 3D, E), indicating that these genes may be involved in hBMSC osteogenic differentiation. Among these genes, 132 were obtained from the intersection of m^6^A and RIP-seq results (Fig. 3D). We selected the top 10 relatively high-expression genes after removing the outliers: FOXD2, JDP2, LRRC32, DRAM1, ZNF839, SLC7A5, THBS1, ZSCAN2, BCR, and HK2. ZNF839 was the gene with the most increased m^6^A modification to be recognized by YTHDF1 and it had a highly enriched and specific m^6^A peak near its translation stop codon (Fig. 3F). We also observed that ZNF839 and its mouse homolog Zfp839 are zinc finger proteins ubiquitously expressed in tissues throughout the body and highly expressed in bone marrow (https://www.ncbi.nlm.nih.gov/gene/55778). Based on the above considerations, we speculated that ZNF839 is an m^6^A-modified gene bound by YTHDF1 and plays a role in the regulation of hBMSC osteogenesis, thus requiring further studies.

### m^6^A “reader” Ythdf1 binds to Zfp839 and promotes translation of m^6^A-modifed Zfp839 mRNA

**Fig. 4:**
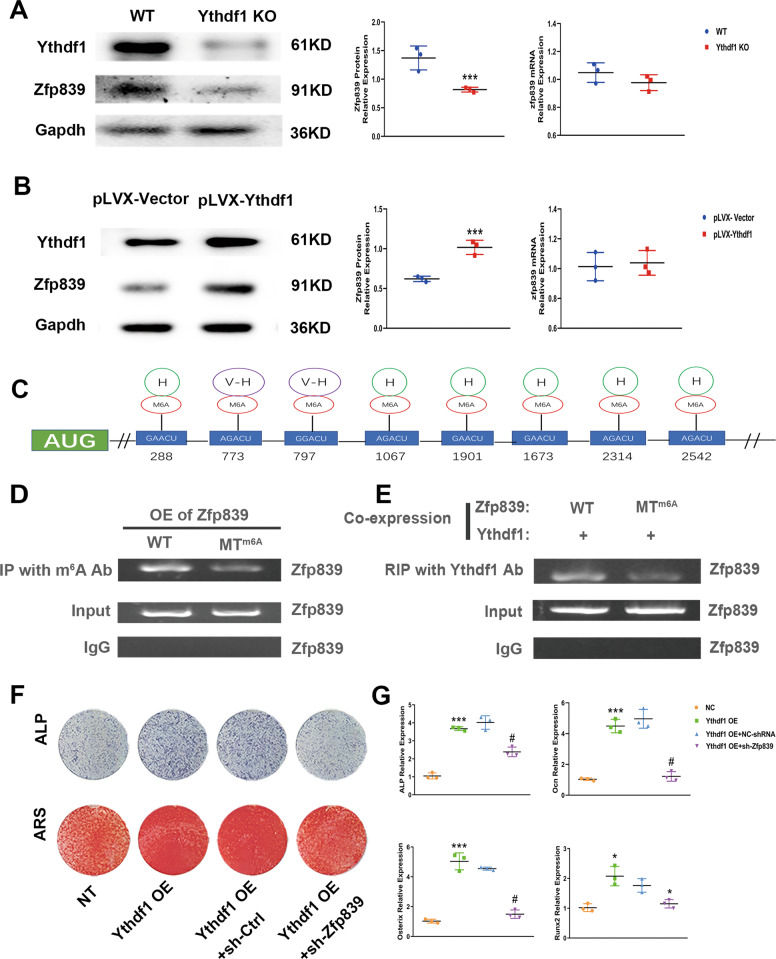
Zfp839 mRNA is modified by m^6^A and binds to the m^6^A reader Ythdf1. **A**, **B** Western blot analysis and RT-PCR analysis showing Zfp839 protein and mRNA levels by Ythdf1 KO or overexpression. ***P < 0.01 versus WT or pLVX-Ythdf1 samples. **C** SRAMP program predicted m^6^A sites in Zfp839 mRNA. H: highly putative predicted m^6^A sites; V-H: very highly putative predicted m^6^A sites. **D** Verification of the m^6^A modification in Zfp839 mRNA. Anti-m^6^A IP failed to pull down Zfp839 mRNA from C3H10T1/2 cells expressing Zfp839 with mutated m^6^A sites (Zfp839-MTm^6^A) compared with Zfp839-WT. **E** Binding of Ythdf1 to Zfp839 mRNA is m^6^A-dependent. RIP using the Ythdf1 Ab failed to pull down Zfp839 mRNA from C3H10T1/2 cells co-expressing Ythdf1 and Zfp839 with mutated m^6^A sites (MTm^6^A) compared with WT Zfp839. **F** ALP and Alizarin Red staining were performed to detect osteogenesis of BMSCs after Ythdf1 overexpression or Ythdf1 overexpression combined with Zfp839 knockdown. **G** mRNA levels of Alp, Ocn, Osterix, and Runx2 were detected after Ythdf1 overexpression or Ythdf1 overexpression combined with Zfp839 knockdown. *P < 0.05, ***P < 0.01 versus NC samples; and *P < 0.05, ^*#*^*P* < 0.01 versus Ythdf1 overexpression samples. Abbreviations: IP, immunoprecipitation; RIP, RNA immunoprecipitation.

We further attempted to confirm whether Zfp839 was regulated by Ythdf1. The mRNA and protein expression levels of Zfp839 in both control and Ythdf1 KO BMSCs were evaluated after 3 days of osteogenesis. Downregulation of the Zfp839 protein level in Ythdf1 KO BMSCs was detected by Western blot analysis. On the contrary, Ythdf1 overexpression in BMSCs showed higher Zfp839 protein levels after 3 days osteogenesis compared with control cells, although the mRNA levels of Zfp839 remained largely unchanged (Fig. 4A, B). These results indicate that the regulation of Zfp839 by Ythdf1 occurred post-transcriptionally.

We then attempted to confirm that Zfp839 expression is regulated by Ythdf1 in an m^6^A dependent manner. First, we searched for the m^6^A motif in Zfp839 using the mammalian m^6^A site predictor SRAMP. Analysis of Zfp839 mRNA with the SRAMP program predicted six High Confidence m^6^A sites at locus 288, 1067, 1901, 1673, 2314, 2542, and two Very High Confidence m^6^A sites at locus 773 and 779 on CDS. For further study, we introduced synonymous mutations at two Very High putative m^6^A sites (Zfp839-Mut) at the same time (Fig. 4C).

Pull-down experiments with the m^6^A antibody were conducted but failed to pull down m^6^A-mutated Zfp839 mRNA from C3H10T1/2 cells expressing Zfp839-MTm^6^A compared with cells expressing Zfp839-WT, indicating a nearly complete loss of m^6^A modifications in Zfp839 mRNA (Fig. 4D). We tried to confirm whether m^6^A-modifed Zfp839 mRNA could be recognized and bound by the m^6^A “reader” Ythdf1. For this purpose, C3H10T1/2 cells were co-transfected with Ythdf1 and Zfp839-WT/MT, and a RIP experiment was performed using an anti-Ythdf1 antibody. The results showed that Zfp839 mRNA was detected in C3H10T1/2 cells co-expressing Ythdf1 and Zfp839-WT; but not in cells co-expressing Ythdf1 and Zfp839-MT (Fig. 4E). These results suggest that the binding of Ythdf1 to Zfp839 mRNA should be m^6^A-dependent.

To verify whether the osteogenic promotion function of Ythdf1 is fulfilled via Zfp839 translation enhancement, we knocked down Zfp839 expression in BMSCs overexpressing Ythdf1. Beforehand, the transfection efficiency of Zfp839 shRNAs was detected. shRNA1 significantly reduced the corresponding Zfp839 mRNA expression and protein levels, whereas shRNA2 and shRNA3 showed no such remarkable effect (Fig. S2A, B). We then conducted the following studies using shRNA1. ALP and Alizarin Red staining showed that enhanced mouse BMSC osteogenesis caused by Ythdf1 overexpression can be abrogated by Zfp839 knockdown (Fig. 4F). PCR conducted with osteogenic-specific genes also showed similar results to those of staining assays (Fig. 4G). All these experiments confirm that the effect of Ythdf1 on BMSC osteogenesis is mediated by its downstream target Zfp839.

To further investigate the relationship of Ythdf1 and Zfp839 in vivo, we conducted immunostaining in bone marrow sections from both WT and Ythdf1 KO mice. Ythdf1 and Zfp839 were detected co-localized in the WT group while they were both scarcely detected in the Ythdf1 KO group, which indicated that Ythdf1 and Zfp839 may be expressively and functionally related in vivo (Fig. S3A).

### Zfp839 promotes BMSCs osteogenesis via interacting with Runx2

**Fig. 5:**
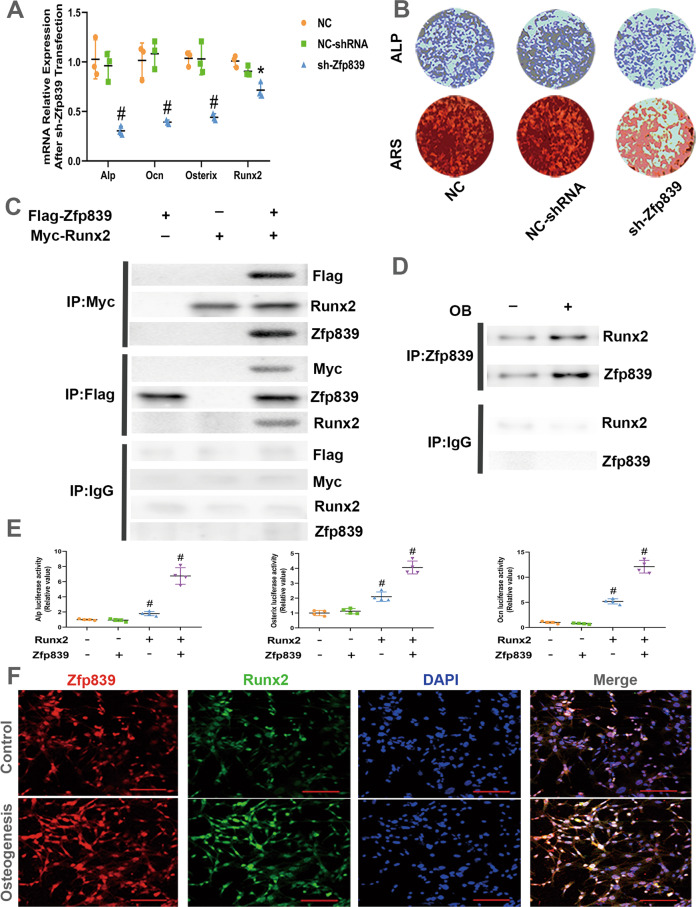
Zfp839 enhanced BMSC osteogenesis via physical interaction with Runx2. **A** mRNA expression levels of Ocn, ALP, Osterix, and Runx2 detected 72 h after osteogenic induction, *P < 0.05, ^*#*^ *P* < 0.01 versus NC or NC-shRNA samples. **B** ALP and Alizarin Red staining were performed to detect the osteogenesis of mouse BMSCs. **C** 293 T cells were transfected with Myc-Runx2 and Flag-Zfp839. Cell lysates were immunoprecipitated with Myc or Flag Abs and then subjected to Western blot analysis with Flag, Myc, Zfp839, or Runx2 Abs. **D** Cell lysates from MC3T3-E1 cells that were subjected to 3 days osteoblastic induction (OB) were immunoprecipitated with the Zfp839 Ab followed by Western blot analysis with the Runx2 Ab. **E** Zfp839 promoted Runx2 transcriptional activity in 293 T cells. 293 T cells were overexpressed with Zfp839 or Runx2 and transfected with the Alp, Osterix, or Ocn luciferase vector. Luciferase activities of Alp, Osterix, and Ocn were detected. All experiments were independently performed in triplicate. Data are presented as the mean ± SD (*n* = 4). (Paired *t* test, n = 4, *P < 0.05 and ^*#*^*P* < 0.01). **F** Immunofluorescence staining for Zfp839 and Runx2 in BMSC osteogenesis induction for 72 h or control cells. Scale bar = 50 µm.

Although we verified the osteogenic function of Ythdf1, with Zfp839 serving as its downstream target, the exact role of Zfp839 in the osteogenic differentiation of BMSCs should still be elucidated. We further conducted gain- and loss-of-function experiments to ascertain the effect of Zfp839 on osteoblast differentiation. Mouse BMSCs transfected with Zfp839 shRNA were incubated with osteogenic medium for 3 days, and knockdown of Zfp839 significantly abrogated the increased expression of osteogenic-specific gene mRNA levels in BMSCs under osteogenic induction (Fig. 5A). The results of ALP and Alizarin Red staining assays also indicate that knockdown of Zfp839 reduced the mineralization of these BMSCs (Fig. 5B). Zfp839 overexpression was achieved by transfection of pLVX-Zfp839 into mouse BMSCs. Transfection efficiency was verified by PCR and Western blot analysis 72 h after pLVX-Zfp839 transfection (Fig. S4A, B). Contrary to the knockdown experiments, Zfp839 overexpression by pLVX-Zfp839 transfection potentiated BMSC osteogenic differentiation, as demonstrated by the elevated expression of osteoblastic genes and the enhanced intensity of ALP and Alizarin Red staining (Fig. S4C, D). All of these results indicate that Zfp839 plays a positive role in BMSC osteogenesis.

We observed that in the loss-/gain-of-function experiments, Zfp839 overexpression or knockdown slightly affected the expression of Runx2 but strongly changed the expression of its downstream osteogenic specific genes (Alp, Osterix, and Ocn) during BMSC osteogenic differentiation. Given that Runx2 is an upstream key transcription factor to upregulate its downstream osteogenic specific genes during BMSC osteogenesis, these results prompted us to speculated that Zfp839 may serve as a co-activator of Runx2 to potentiate its transcriptional activity rather than serving as an upstream factor to directly promote Runx2 expression.

Furthermore, IP experiments were performed by co-transfecting Flag-tagged Zfp839 or Myc-tagged Runx2 into HEK-293T cells, after which the proteins were extracted and subjected to IP with anti-Myc or anti-Flag antibodies. As shown in Fig. 5C, Zfp839 can directly interact with Runx2. Additionally, an IP assay indicated that endogenous Zfp839 can interact with Runx2 in MC3T3-E1 cells (Fig. 5D).

We further constructed reporter plasmids containing osteoblast-specific gene promoter regions and these plasmids were co-transfected with Runx2 into 293 T cells. A luciferase reporter assay indicated that the overexpression of Zfp839 alone did not influence luciferase activity. Overexpression of Runx2 alone activated the Alp, Osterix, and Ocn promoters. However, when Zfp839 was co-transfected with Runx2, the transcriptional activity of Runx2 was significantly enhanced, as indicated by the dramatically increased luciferase activity under the promoters of Alp, Osterix, and Ocn (Fig. 5E). To investigate the role of Zfp839 and Runx2 in the process of BMSC osteogenesis, we also conducted immunofluorescence staining on BMSCs that underwent osteogenic induction for 72 h. Enhanced fluorescence intensities of Zfp839 and Runx2 in osteogenesis-induced BMSCs were detected compared with the control cells, which suggested that Zfp839 and Runx2 may be part of the same regulatory complex colocalized in the nucleus of BMSCs involved in osteogenesis (Fig. 5F). Altogether, these data show that Zfp839 regulates osteoblast-specific gene expression through interacting with Runx2.

### Deletion of Ythdf1 leads to low bone mass in vivo

**Fig. 6:**
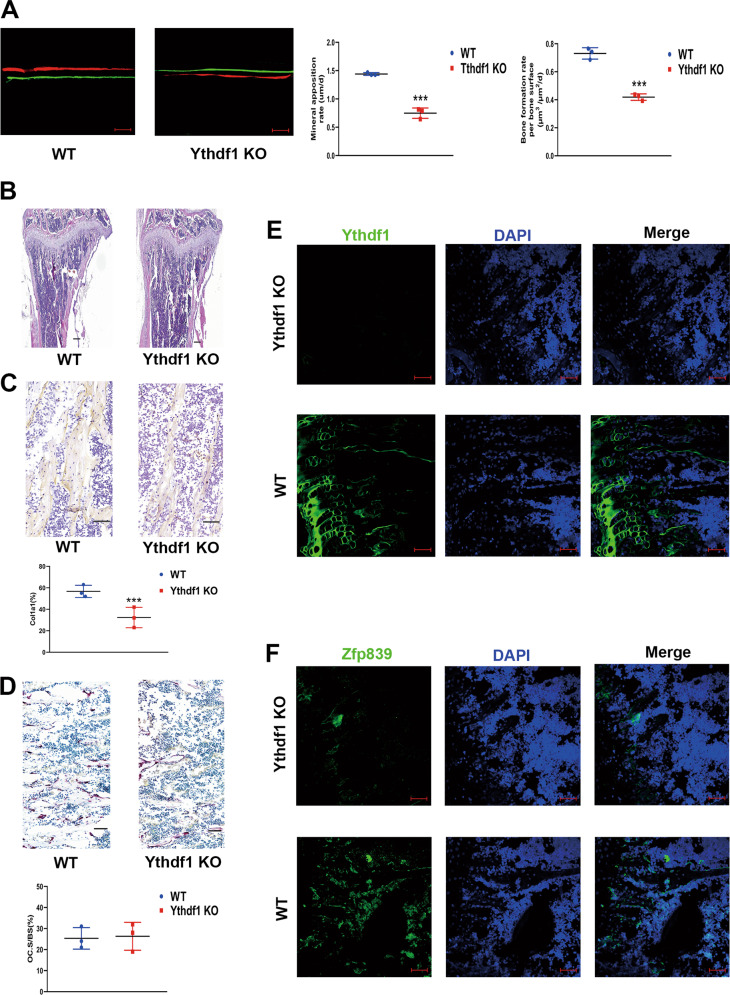
Ythdf1 potentiates osteoblast differentiation in vivo. **A** Representative images of calcein and Alizarin Red S-labeled bone formation with MAR and BFR/BS analysis in WT and Ythdf1 KO mice; scale bar = 50 µm. Quantification analysis was performed using ImageJ software. (*n* = 3), ***P < 0.01. **B** H&E staining of the murine tibia from 3-month-old male WT and Ythdf1 KO mice. Scale bar = 200 µm. **C** Immunohistochemical staining of Col1a1 in proximal tibia metaphysis from WT and Ythdf1 KO mice. Scale bar = 20 µm. Quantification analysis was performed using ImageJ software. (*n* = 3), ***P < 0.01. **D** Representative TRAP staining images of the metaphysis and quantitative analysis of the ratio of OC surface to bone surface (OC.S/BS) from WT and Ythdf1 KO mice. scale bar = 50 µm. **E**, **F** Ythdf1 and Zfp839 expression detected by immunofluorescent assays in both groups; scale bar = 50 µm.

To further verify the role of Ythdf1 in bone metabolism in vivo, we found that Ythdf1 KO mice demonstrated both a decreased mineral apposition rate (MAR) and bone formation rate (BFR) compared with their control WT littermates by fluorescent double labelling of the mineralizing front with calcein and Alizarin red S (Fig. 6A), indicating a lower bone formation rate in Ythdf1 KO mice than in WT controls.

In addition, we detected reduced bone mass in hematoxylin and eosin (H&E) staining (Fig. 6B) as well as decreased collagen type I alpha 1 chain (Col1a1) in immunohistochemical sections from Ythdf1 KO mice (Fig. 6C). Tartrate-resistant acidic phosphatase (TRAP) staining was also conducted, and no significant difference in stained area quantification was detected between the two groups (Fig. 6D), indicating that Ythdf1 did not influence osteoclast activity nor bone resorption. Furthermore, the immunofluorescence assay scarcely detected Ythdf1 expression in the KO group, and the expression of Zfp839 was also reduced (Fig. 6E, F).

These in vivo results also indicate that the retarded bone formation was possibly due to the diminished ability of the Ythdf1-lacking BMSCs to undergo osteogenic differentiation.

## Discussion

M^6^A modification plays important roles in physiological or pathological processes including hBMSC fate determination [12]. Most studies have focused on m^6^A modification via addition and deletion by methyltransferases or demethylases [13, 14]. However, m^6^A modification determines the fate of target mRNA by recruiting different m^6^A “readers”, and the function of m^6^A-modified mRNAs is ultimately interpreted by these “readers”. For example, YTHDC1 facilitates the nuclear export of m^6^A-modified RNAs into the cytoplasm [15]. In the cytoplasm, m^6^A-containing transcripts are sorted for degradation when bound by YTHDF2, YTHDF3, or YTHDC2 [16–18], whereas IGF2BP1/2/3 and PRRC2A help to stabilize m^6^A-modified transcripts [19, 20]. YTHDF1 is an m^6^A “reader” widely distributed in the body, including the bone marrow, and plays important roles in cancer progression, neuronal development, and regeneration, as well as stem cell proliferation, differentiation, and aging [21–23]. Its function via m^6^A-modified mRNAs recognization and further translation promotion was reported by several studies [21, 22, 24]. However, the understanding of how m^6^A modification is recognized, and its function in BMSC osteogenic differentiation is still limited: what is the “reader”, the downstream targets and their roles in BMSC osteogenesis are all unknown. In the present study, we observed that YTHDF1 played a pivotal role in hBMSC osteogenic differentiation. Its knockdown reduced the osteogenesis of hBMSCs in vitro. In vivo, Ythdf1 KO mice showed decreased bone mass compared with their WT littermates. We identified ZNF839 as a downstream target of YTHDF1 via Me-RIP-Seq and RIP-Seq. Although the physiological or pathological role of ZNF839 in organisms has not been elaborated in the literature, its zinc-finger structure prompts us to speculate that it may serve as a transcription factor and play a role in hBMSC osteogenesis; our loss-/gain-of-function experiments also verified this osteogenesis potentiation function.

Runx2 is a key transcription factor essential for BMSC osteoblast commitment, its expression is regulated by other transcription factors and its transcriptional activity is largely controlled by several co-factors. Plzf and Msx2 serve as upstream factors of Runx2 to promote BMSCs osteoblast differentiation [25]. CBFB, MOZ, MORF, and TAZ functioned as transcriptional co-activators of Runx2; Twist, Stat1, Smad3, KLF4, and TLE reduced Runx2 transcriptional activity as co-repressors [26, 27]. In this study, the results from ALP and ARS assay and PCR analysis indicated that Zfp839 can potentiate BMSC osteogenesis. However, Runx2 expression was less disturbed by Zfp839 knockdown or overexpression compared with other osteogenesis-related genes. Thus, Zfp839 might function as a co-transcription factor of Runx2 and control its transcriptional activity rather than as an upstream transcription factor that regulates its expression. The results of IP experiments and luciferase assay confirmed the physical interaction of Zfp839 with Runx2 and the increased luciferase activities of the promoters of downstream osteogenic-specific genes. All these experiments indicate that Zfp839 may serve as a co-activator of Runx2.

The activity of transcription factors can be altered by their epigenetic modification states, such as DNA methylation, histone acetylation, and methylation. Osterix remains in a methylated state in undifferentiated BMSCs, whereas during the osteogenic differentiation, it undergoes demethylation modification, and its transcription activity was activated [28]; HDAC3 could also directly bind to Runx2 and subsequently inhibit osteoblast differentiation [29]. Histone demethylase JARID1B demethylates H3K4me3 on the P1 promoter of RUNX2, thereby inhibiting the osteogenic differentiation of BMSCs [30]. Different from the above-mentioned epigenetic modifications targeting transcription factor activity, m^6^A modification controls the protein expression level of its downstream targets. In this study, the zinc-finger protein ZNF839/Zfp839 was post-transcriptionally controlled by Ythdf1 in an m^6^A-dependent manner. Moreover, it served as a novel co-activator that potentiated BMSC osteogenesis via interacting with the master transcription factor Runx2. This discovery elucidates a new layer of epigenetic modification of transcription factors that are involved in BMSC osteogenesis control.

This study depicted a dynamic and multi-dimensional mechanism of m^6^A modification in modulating transcription factor expression, function, and further hBMSC osteogenic differentiation, showing a novel link between YTHDF1 mediated posttranscriptional modification and the transcription regulatory network in bone metabolism. These findings offer a new approach to dissect the molecular mechanism of BMSC osteogenesis, and provide a potential target for the treatment of metabolic bone diseases.

## Materials and methods

### Construction of Ythdf1 knockout mice

This study was approved by the Institutional Ethics Committee of Xinhua Hospital affiliated to Shanghai Jiaotong University School of Medicine (XHEC-NSFC-2019-153). All applicable institutional and national guidelines for the care and use of animals were followed. Ythdf1 CRISPR–Cas9 knockout (Ythdf1 KO) mice were constructed as previously reported [31], the KO mice were gifts from the School of Life Science and Technology, Shanghai Tech University. The genotype of all progeny was confirmed by PCR analysis of the DNA extracted from tail biopsies. All procedures were performed in accordance with NIH guidelines for animal experimentation. Five animals were housed per cage under standard laboratory conditions (21 ± 2 °C, humidity 60% ± 10%, and a 12 h/12 h dark/light cycle). All the animals used in the present study were male mice with the same C57BL/6 genetic background. The WT and Ythdf1 KO mice from the same litter were matched and were randomly selected for further experiments. For each experiment, animals were transferred to the experimental room 30 min prior to the experiment to acclimate them to the environment.

### µCT and histomorphometric analyses

The tibias from WT and Ythdf1 KO mice were skinned and fixed with 4% paraformaldehyde and then scanned using an 80 µCT system (Scanco Medical, Bassersdorf, Switzerland) at a spatial resolution of 5 µm (55 kV, 114 mA, and 500 ms integration time). The proximal metaphysis between 1.5 and 4 mm distal to the growth plate was set as the region of interest. Three-dimensional and two-dimensional images were generated using the CTvol program (Skyscan). Micro-architectural parameters, including BV/TV, Tb.Th, Tb.N, and Tb.Sp, were obtained and analyzed.

To evaluate the BFR in vivo, 12-week-old WT and Ythdf1 KO mice were intraperitoneally injected with green fluorescent calcein (5 mg/kg, Sigma-Aldrich) and Alizarin Red S (40 mg/kg, Sigma-Aldrich) at 3 and 10 days before euthanasia. The tibias were then dissected, fixed, dehydrated, and embedded in methyl methacrylate resin, after which the tissue sections were observed under a laser-scanning microscope (LSM5 PASCAL; Carl Zeis).

### Lentiviral Packaging

pLVX-IRES-puro, pLVX-IRES-puro-YTHDF1, pLVX-IRES-puro-Ythdf1, pLVX-IRES-puro-ZNF839, and pLVX-IRES-puro-Zfp839 were transfected into the HEK293T viral packaging cell line together with pSPAX2 and pMD 2.G. Exactly 48 h after transfection, the cells were harvested for RT-PCR or Western blot analysis to verify the packaging efficiency.

The lentiviral knockdown vectors pLKO.1-eGFP for YTHDF1, ZNF839 and Runx2 were constructed, and the virus was prepared according to previous publications. Table 1 lists the target sequences of the shRNAs for YTHDF1, Zfp839, and Runx2.

**Table 1:** shRNAs for YTHDF1 and Zfp839.

ShRNAs	Target Sequences
Sh1 YTHDF1	F: 5’-GCUCCAUUAAGUACUCCAUTT-3’ R: 5’-AUGGAGUACUUAAUGGAGCTT-3’
Sh2 YTHDF1	F: 5’GGAUACAGUUCAUGACAAUTT-3’
	R: 5’-AUUGUCAUGAACUGUAUCCTT-3’
Sh3 YTHDF1	F: 5’-CCUCCACCCAUAAAGCAUATT-3’
	R: 5’-UAUGCUUUAUGGGUGGAGGTT-3’
Sh1 Zfp839	F: 5’-GCAGGAACUUGAAGCUAUUTT-3’
	R: 5’-AAUAGCUUCAAGUUCCUGCTT-3’
Sh2 Zfp839	F: 5’-GCACGUACUGAGAAGUCAATT-3’
	R: 5’-UUGACUUCUCAGUACGUGCTT-3’
Sh3 Zfp839	F: 5’-GGAAGAUGACAGUGUCGUUTT-3’
	R: 5’-AACGACACUGUCAUCUUCCTT-3’

The sequences of shRNAs targeting YTHDF1 and Zfp839.

### Immunohistochemical staining

The dissected tibia samples were fixed in 4% polyoxymethylene for 24 h, decalcified in 12.5% ethylenediaminetetraacetic acid (EDTA) (pH = 7.0) for 21 days, and then embedded in paraffin and sectioned for staining. Serial sections (6 µm) of the tibias were incubated with primary rabbit polyclonal anti-Col1a1 (1:500 dilution) overnight at 4 °C.

A horseradish peroxidase–streptavidin detection system (Dako, Glostrup, Sweden) was used to detect immunoreactivity, hematoxylin was used to counterstain the nuclei. Immunohistochemical analysis was performed with a light microscope (Leica, Germany).

### Immunofluorescence assay

Paraffin-embedded sections (5 µm thick) of the decalcified tibia from WT and Ythdf1 KO mice were incubated overnight at 4 °C with antibodies (Abs) against Ythdf1 and Zfp839 respectively, and covered with 4’,6-diamidino-2-phenylindole (DAPI) to visualize the nuclei. The primary antibodies were detected using a fluorescein isothiocyanate-conjugated anti-rabbit IgG secondary Ab. The stained sections were observed under a laser confocal microscope (Cell Observer, ZEISS, Germany).

A total of 2 × 10^5^ BMSCs were seeded in a 30-mm confocal dish. After 72 h osteogenic induction, the cells were fixed in 4% paraformaldehyde, permeabilized in 0.2% Triton X-100 and probed with specific primary antibodies for Zfp839 (LSBio, LS-C658560, Seattle, WA, USA) and Runx2 (Abcam, ab76956, Cambridge, UK). The primary Abs were then detected using matching anti-mouse or anti-rabbit IgG secondary Abs. Then cells were also co-stained with DAPI to detect nuclei and observed under a laser confocal microscope (Cell Observer, ZEISS, Germany).

### BMSC culture and osteogenic differentiation

To isolate mouse BMSCs, we first extracted bone marrow cells from the femurs and tibias of Ythdf1-KO mice by flushing the cells with a modified Eagle’s medium (Invitrogen, Carlsbad, CA, USA) supplemented with 10% fetal bovine serum (#16000-044; Gibco, AUS), 100 U/mL penicillin G, and 100 mg/L streptomycins (#SV30010; HyClone, Logan, UT, USA). The bones were crushed with a pestle and treated with collagenase (Wako, Osaka, Japan), after which BMSCs were plated on 10 cm culture plates and incubated in 5% CO_2_ at 37 °C. The medium was changed twice every week to remove unattached cells until confluence was achieved.

hBMSCs were obtained from nine young healthy male individuals (age range: 20–30 years old, average: 26.4 years) who underwent traumatic femoral or tibia shaft fracture treatment by intramedullary nailing and nine aged osteoporotic male patients (age range: 70–80 years old, average: 75.2 years) who received total hip arthroplasty after providing written consent. Cell extraction and passage were performed as previously described [32]. Briefly, bone marrow blood from donors was filtered through a 100 µm nylon mesh cell strainer and then incubated in basal medium (BM) [low glucose Dulbecco’s modified Eagle’s medium (#SH30021.01; HyClone, Logan, UT, USA) supplemented with 10% fetal bovine serum (#16000-044; Gibco, AUS), 100 U/mL penicillin G, and 100 mg/L streptomycin (#SV30010; HyClone, Logan, UT, USA)] at 37 °C in a humidified atmosphere containing 5% CO_2_. The established hBMSCs were then used for subsequent experiments at passages 3 to 7.

For osteogenic induction, BMSCs were seeded in six-well plates and cultured in osteogenic medium (BM supplemented with 1 nM dexamethasone (#D4902; Sigma-Aldrich), 50 mM ascorbic acid (#A4403; Sigma-Aldrich), and 20 mM β-glycerolphosphate (#G9891; Sigma-Aldrich). The culture medium was changed every 3 days.

### ALP and Alizarin red staining

For ALP staining, cells were washed thrice with phosphate-buffered saline (PBS) and fixed with 4% polyoxymethylene after 7 days of osteogenic induction. Then, the cells were incubated with a 5-bromo-4-chloro-3-indolyl phosphate/nitro blue tetrazolium staining solution. After a 15 min incubation at 37 °C, the cell layer was washed thrice with deionized water and observed under a digital camera. For Alizarin Red staining, the cells were fixed after 14 days of osteogenic induction with 4% polyoxymethylene for 15 min and stained with Alizarin Red S solution (Sigma-Aldrich) for 15 min until they were orange-red in color. After staining, the cells were washed thrice with deionized water and observed under a digital camera.

### Quantitative RT-PCR

Total RNA of cultured cells was extracted using TRIzol reagent (Invitrogen, CA, USA), and cDNA was synthesized with PrimeScript RT Master Mix cDNA Synthesis Kit (Takara, Japan) using 2 µg extracted RNA per sample. RT-PCR was performed with a Roche LC 480 system using SYBR1 Premix (TaKaRa, Inc., Dalian, China) following the manufacturer’s instructions. Relative gene expression was normalized to glyceraldehyde 3-phosphate dehydrogenase (GAPDH), and the data were analyzed using the Ct (2^−ΔΔCt^) method. Table 2 lists the primers used in the study.

**Table 2:** Primer sequences for RT-qPCR.

Gene	Primer Sequences
human GAPDH	F: 5’-AGGTCGGTGTGAACGGATTTG-3’ R: 5’-GGGGTCGTTGATGGCAACA-3’
human YTHDF1	F: 5’-ACCTGTCCAGCTATTACCCG-3’ R: 5’-TGGTGAGGTATGGAATCGGAG-3’
human ZNF839	F: 5’-TCCTACCGACCACAATCCAG-3’ R: 5’-CAAGCGGCTGTACCCTGAG-3’
human ALP	F: 5’-ACCACCACGAGAGTGAACCA-3’ R: 5’-CGTTGTCTGAGTACCAGTCCC-3’
human COL1A1	F: 5’-GAGGGCCAAGACGAAGACATC-3’ R: 5’-CAGATCACGTCATCGCACAAC-3’
human OCN	F: 5’-GACAAGTCCCACACAGCAACT-3’ R: 5’-GGACATGAAGGCTTTGTCAGA-3’
human OSTERIX	F: 5’-CCTCTGCGGGACTCAACAAC-3’ R: 5’-AGCCCATTAGTGCTTGTAAAGG-3’
human RUNX2	F: 5’-TGGTTACTGTCATGGCGGGTA-3’ R: 5’-TCTCAGATCGTTGAACCTTGCTA-3’
mouse Gapdh	F: 5’- AGGTCGGTGTGAACGGATTTG-3’ R: 5’- TGTAGACCATGTAGTTGAGGTCA-3’
mouse Ythdf1	F: 5’-ACAGTTACCCCTCGATGAGTG-3’ R: 5’-GGTAGTGAGATACGGGATGGGA-3’
mouse Zfp839	F: 5’-CCAGTCCCTTTGAAGAGAGCC-3’ R: 5’-GTCCAGATCGAGTTCTTACCCT-3’
mouse Alp	F: 5’-AGGGCAATGAGGTCACATCC-3’ R: 5’-GCATCTCGTTATCCGAGTACCAG-3’
mouse Col1a1	F: 5’-TGTGTGCGATGACGTGCAAT-3’ R: 5’-GGGTCCCTCGACTCCTACA-3’
mouse Ocn	F: 5’-GAACAGACAAGTCCCACACAGC-3’ R: 5’-TCAGCAGAGTGAGCAGAAAGAT-3’
mouse Osterix	F: 5’-CCTCTGCGGGACTCAACAAC-3’ R: 5’-AGCCCATTAGTGCTTGTAAAGG-3’
mouse Runx2	F: 5’-TGTTCTCTGATCGCCTCAGTG-3’ R: 5’-CCTGGGATCTGTAATCTGACTCT-3’

Real-Time PCR primer sequences of YTHDF1, Zfp839 and osteoblast-specific genes used in this study. (ALP, alkaline phosphatase; COL1A1, Type I collagen; OCN, osteocalcin; GAPDH, glyceraldehyde-3-phosphate dehydrogenase).

### Western blot

After being washed with ice-cold Dulbecco’s PBS, cells were lysed in RIPA buffer (Biocolors, R0095, Shanghai, China) containing 1% PMSF (Meilunbio, MA0001, Dalian, China). Approximately 20 ug of proteins were resolved on 10% SDS-PAGE gels (Bio-Rad, Richmond, CA) and transferred to polyvinylidene difluoride membranes (Merch, ISEQ00010). The membranes were blocked with Tris-buffered saline containing 5% non-fat milk and 0.1% Tween-20 for 1 h. Then, they were sequentially incubated with primary and secondary antibodies. The immunoblots were visualized using an enhanced chemiluminescence detection system (Millipore, Billerica, MA, USA). The primary antibodies used for Western blot analysis included YTHDF1 (Proteintech,17479-1-AP, Beijing, China), ZNF839 (LSBio, LS-C658560, Seattle, WA, USA), and GAPDH (CST, 5174, Danvers, MA, USA). A horseradish peroxidase-labeled secondary antibody was also used in our work.

### Luciferase assay

Briefly, ALP, Osterix, and OCN gene promoter-luciferase reporters; flag-tagged Zfp839; Myc-tagged mouse Runx2; and empty vector pcDNA3.1 plasmid were transfected into HEK-293T cells using Lipofectamine 2000 (Life Technologies, USA). After 24 h of incubation, the cells were washed twice with ice-cold PBS and lysed. Then, luciferase activity was measured using a luciferase reporter assay system (Promega, USA) on a multi-plate reader (Bio-Tek Instruments, USA) in accordance with the manufacturer’s instructions. All data are presented as the mean ± standard deviation (SD) of three independent experiments.

### MeRIP-Seq

The m^6^A RIP was performed with GenSeqTM m^6^A-MeRIP Kit (GenSeq Inc., China) following the manufacturer’s instructions. Briefly, TRIzol reagent (Invitrogen) was used for the isolation of total RNA from hBMSCs, and random mRNA fragments (−200 nt) were generated by using an RNA fragmentation reagent. Samples were then incubated with anti-m^6^A-pAb at 4 °C for 2 h. The mixture was immunoprecipitated by incubation with Pierce™ Protein A/G Magnetic Beads at 4 °C for 3 h. Methylated fragments were then eluted from the beads with m^6^A and precipitated with ethanol after extensive washing. Both the input sample without IP and the m^6^A IP samples were used for RNA-Seq library generation using the NEBNext^®^ Ultra II Directional RNA Library Prep Kit (New England Biolabs, Inc., USA). The quality of the library was evaluated using a BioAnalyzer 2100 system (Agilent Technologies, Inc., USA), and library sequencing was performed on an Illumina HiSeq instrument with 150 bp paired-end reads.

### RIP-Seq

RIP was performed using the EZ-Magna RIP™ RNA-Binding Protein Immunoprecipitation Kit (Millipore) following the manufacturer’s protocol. Briefly, hBMSCs transfected with pLVX-YTHDF1 and the pLVX-Vector were homogenized in lysis buffer supplemented with protease and phosphatase inhibitor cocktail (Thermo Fisher Scientific) and an RNase inhibitor (Promega). The beads were incubated with the YTHDF1 antibody (Proteintech) or normal IgG at room temperature for 30 min and then were incubated with lysate supernatant in IP buffer supplemented with EDTA overnight at 4 °C. After extensive washing with IP buffer, the beads were treated with proteinase K for 30 min at 55 °C with occasional shaking. RNA was purified from the supernatant using TRIzol reagent following the manufacturer’s instructions. rRNA was removed from the immunoprecipitated RNA and input RNA samples (New England Biolabs, Inc., Massachusetts, USA). RNA libraries were constructed using rRNA-depleted RNA with NEBNext^®^ Ultra™ II Directional RNA Library Prep Kit (New England Biolabs, Inc., Massachusetts, USA) following the manufacturer’s instructions. The libraries were controlled for quality and quantified using a BioAnalyzer 2100 system (Agilent Technologies, Inc., USA). Library sequencing was performed on an Illumina HiSeq instrument with 150 bp paired-end reads.

### Statistical analysis

All data are presented as the mean ± standard error of the mean. All experiments were conducted with a minimum of three independent biological replicates. Graphs were generated with GraphPad Prism 6.0, and statistical analyses were performed using SPSS software (version 16.0; SPSS, Inc., Chicago, IL). Significant differences were analyzed by unpaired two-tailed Student’s *t* test for comparison between two groups and one-way ANOVA followed by Tukey’s post hoc test for multiple comparisons. All tests were two-sided with a *P* value of 0.05 as the boundary of statistical significance: *P < 0.05, ***P < 0.01.

## Supplementary information

Supplemental figure legend

Checklist

Fig.S1 Loss- and gain-of-Ythdf1 function on osteogenesis in mouse BMSCs.

Fig.S2 Zfp839 knockdown efficiency verification.

Fig.S3 Co-localization of Ythdf1 and Zfp839 in bone marrow.

Fig.S4 Zfp839 overexpression potentiates mouse BMSCs osteogenesis.

## Data Availability

The microarray data is available at https://share.weiyun.com/xcB3waFn.
