# Supplementary material for: The m6A “reader” YTHDF1 promotes osteogenesis of bone marrow mesenchymal stem cells through translational control of ZNF839
Source: Cell Death Dis. 2021 Nov 12;12(11):1078. doi: 10.1038/s41419-021-04312-4 (PMC8590051; doi:10.1038/s41419-021-04312-4)
Supplement: Supplementary file 1 — Supplemental figure legend [file 41419_2021_4312_MOESM1_ESM.pdf]

Fig. S1 Loss- and gain-of-Ythdf1 function on osteogenesis in mouse BMSCs.

(A and B) Ythdf1 mRNA and protein levels detected 72 h after transfection of pLVX-Ythdf1. *** *P* <0.01 compared with pLVX-Vector. **(C)** ALP and Alizarin Red staining to detect osteogenesis of BMSCs after pLVX-Ythdf1 transfection for 7 or 14 days. **(D)** mRNA expression levels of Alp, Ocn, Osterix and Runx2 detected 72 h after osteogenic induction. **P* <0.05, ****P* <0.01 compared with the NC and pLVX-Vector groups. **(E)** ALP and Alizarin Red staining of WT and Ythdf1 KO cells. **(F)** mRNA expression of osteoblast-specific genes detected in WT and Ythdf1 KO cells 72 h after osteogenic induction (Paired t test, n=3, **P* <0.05 and ****P* <0.01). All experiments were independently performed in triplicate.

Fig. S2 Zfp839 knockdown efficiency verification.

Three shRNAs were generated to suppress Zfp839 expression. **(A and B)** Zfp839 mRNA and protein expression levels detected 72 h after shRNA transfection. (Paired t test, n=3, **P* <0.05, ****P* <0.01 compared with the NC and NC-shRNA groups). All experiments were independently performed in triplicate.

Fig. S3 Co-localization of Ythdf1 and Zfp839 in bone marrow.

(A) Immunofluorescence staining showing that Ythdf1 and Zfp839 co-localized in bone marrow in WT group, which cannot be shown in Ythdf1 KO group. Scale bar = 20 μm.

Fig. S4 Zfp839 overexpression potentiates mouse BMSCs osteogenesis.

(A and B) Zfp839 mRNA expression and protein levels detected 72 h after transfection of pLVX-Zfp839. (Paired t test, n=3, ****P* <0.01 compared with pLVX-Vector). (C) mRNA expression of osteogenic-specific genes detected 72 h after osteogenic induction of Zfp839 overexpression, (Paired t test, n=3, **P* <0.05, $^{\#}P$ <0.01 versus NC or pLVX-Vector samples). (D) ALP and Alizarin Red staining performed to detect the osteogenesis of mouse BMSCs after Zfp839 overexpression. All experiments were independently performed in triplicate.
