# Supplementary figures and images for: The m6A “reader” YTHDF1 promotes osteogenesis of bone marrow mesenchymal stem cells through translational control of ZNF839
Source: Cell Death Dis. 2021 Nov 12;12(11):1078. doi: 10.1038/s41419-021-04312-4 (PMC8590051; doi:10.1038/s41419-021-04312-4)

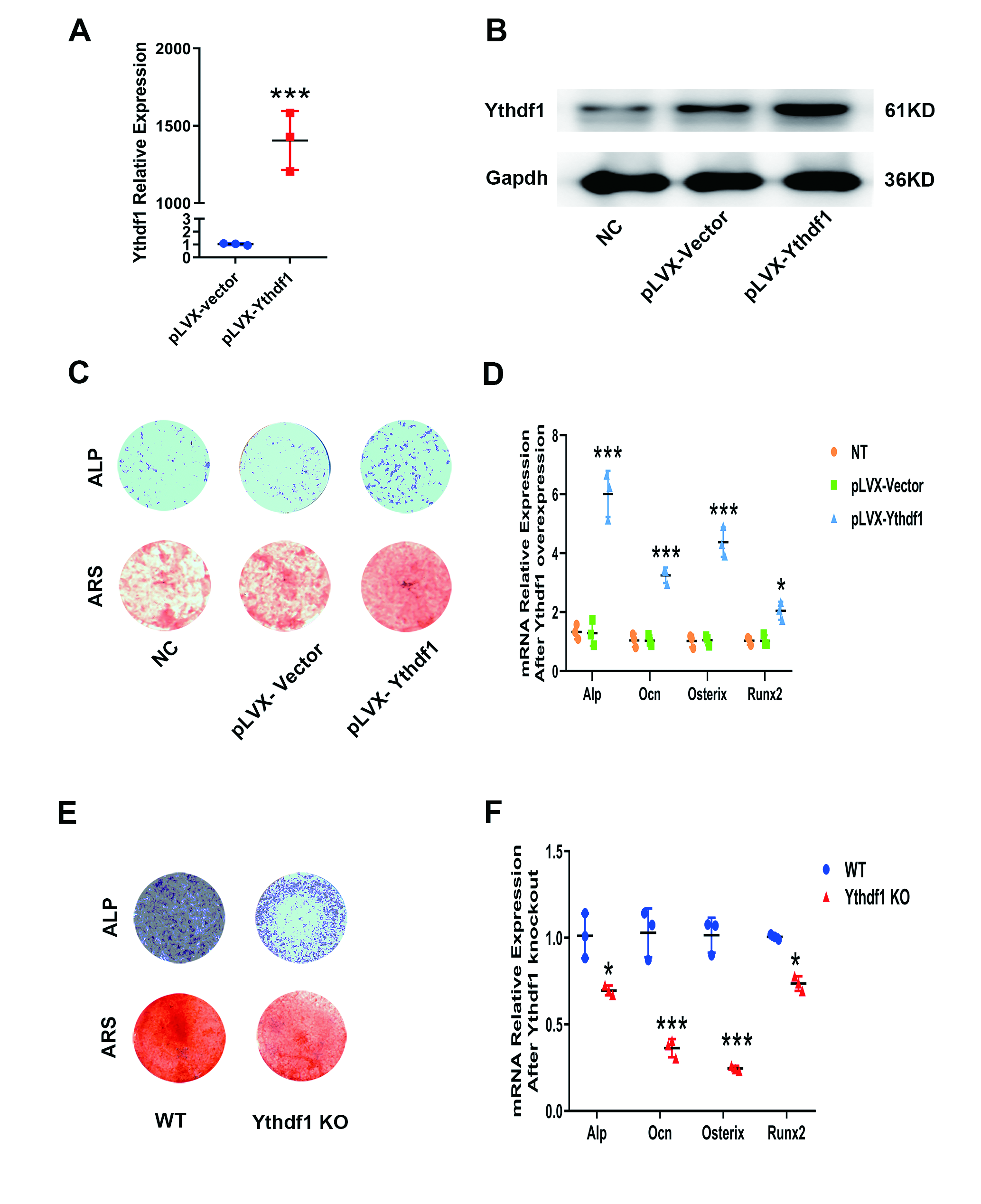

Supplement: Supplementary file 3 — Fig.S1 Loss- and gain-of-Ythdf1 function on osteogenesis in mouse BMSCs. [file 41419_2021_4312_MOESM3_ESM.tif]

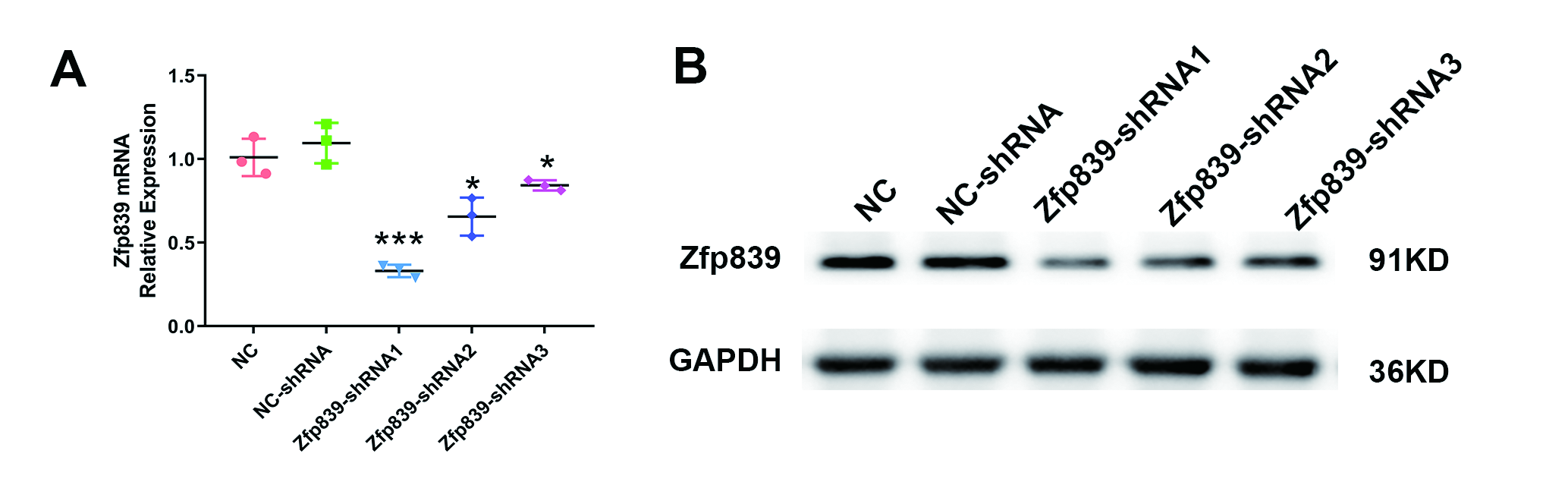

Supplement: Supplementary file 4 — Fig.S2 Zfp839 knockdown efficiency verification. [file 41419_2021_4312_MOESM4_ESM.tif]

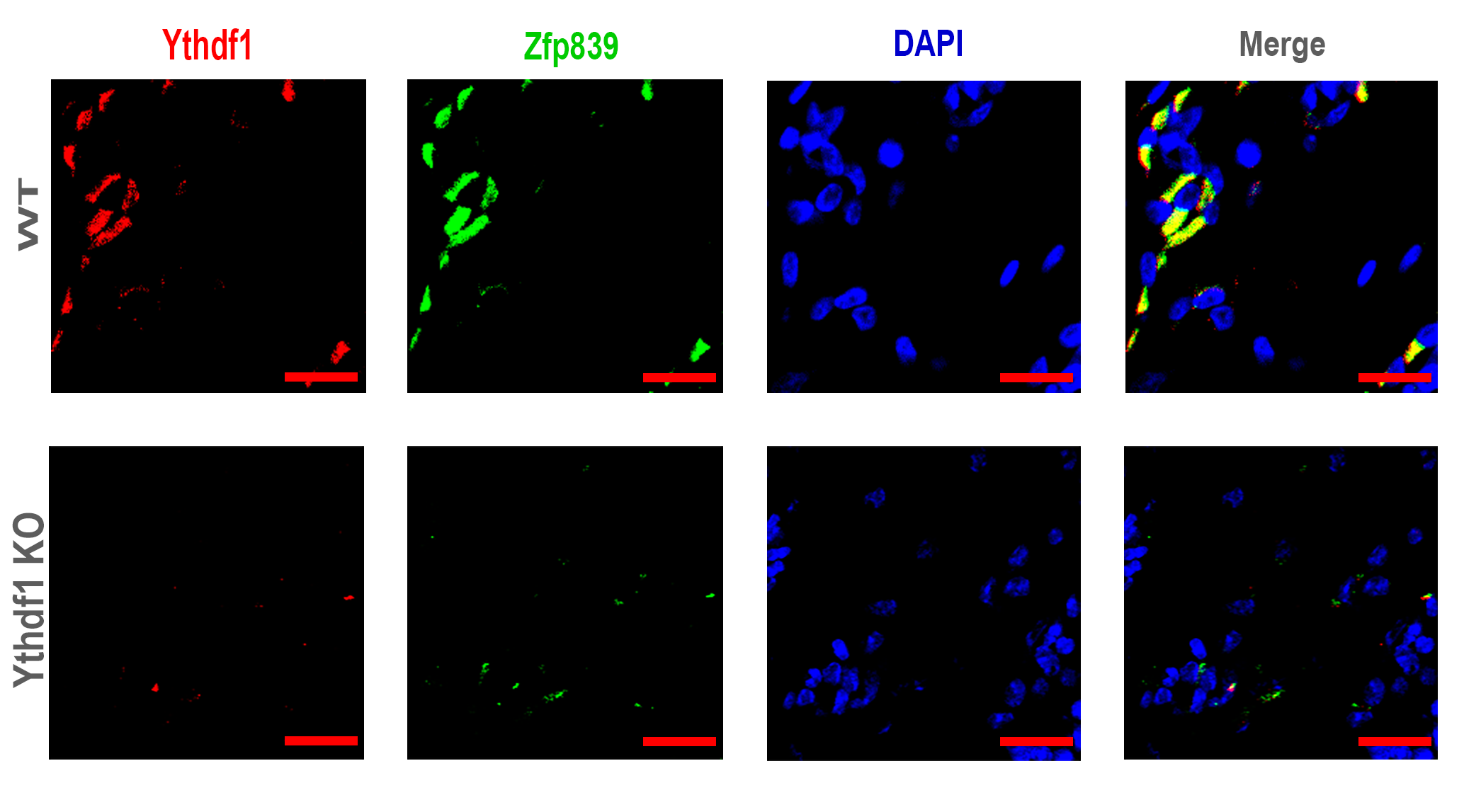

Supplement: Supplementary file 5 — Fig.S3 Co-localization of Ythdf1 and Zfp839 in bone marrow. [file 41419_2021_4312_MOESM5_ESM.tif]

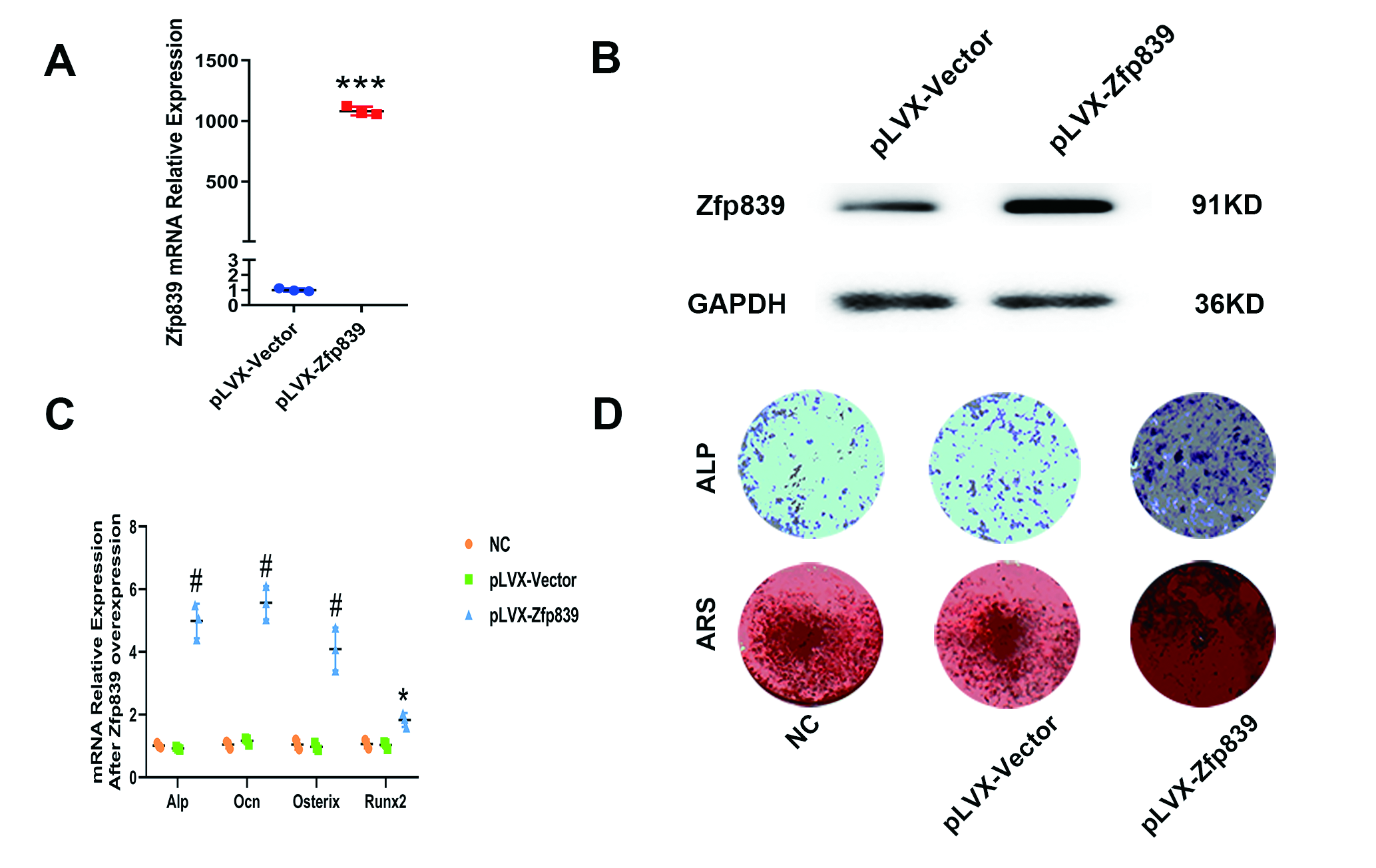

Supplement: Supplementary file 6 — Fig.S4 Zfp839 overexpression potentiates mouse BMSCs osteogenesis. [file 41419_2021_4312_MOESM6_ESM.tif]
